# GK-1 Improves the Immune Response Induced by Bone Marrow Dendritic Cells Loaded with MAGE-AX in Mice with Melanoma

**DOI:** 10.1155/2014/158980

**Published:** 2014-10-29

**Authors:** Gabriela Piñón-Zárate, Miguel Ángel Herrera-Enríquez, Beatriz Hernández-Téllez, Katia Jarquín-Yáñez, Andrés Eliú Castell-Rodríguez

**Affiliations:** Laboratorio de Inmunoterapia e Ingeniería de Tejidos, Departamento de Biología Celular y Tisular, Facultad de Medicina, Universidad Nacional Autónoma de México Edificio A, Sexto Piso, Ciudad Universitaria, Avenida Universidad No. 3000, Ciudad de México 04510DF, Mexico

## Abstract

The aim of dendritic cell (DC) vaccination in cancer is to induce tumor-specific effector T cells that may reduce and control tumor mass. Immunostimulants that could drive a desired immune response are necessary to be found in order to generate a long lasting tumor immune response. GK-1 peptide, derived from *Taenia crassiceps*, induces not only increase in TNF*α*, IFN*γ*, and MCP-1 production in cocultures of DCs and T lymphocytes but also immunological protection against influenza virus. Moreover, the aim of this investigation is the use of GK-1 as a bone marrow DCs (BMDCs) immunostimulant targeted with MAGE antigen; thus, BMDC may be used as immunotherapy against murine melanoma. GK-1 induced in BMDCs a meaningful increment of CD86 and IL-12. In addition, the use of BMDCs TNF*α*/GK-1/MAGE-AX induced the highest survival and the smallest tumors in mice. Besides, the treatment helped to increase CD8 lymphocytes levels and to produce IFN*γ* in lymph nodes. Moreover, the histopathological analysis showed that BMDCs treated with GK-1/TNF*α* and loaded with MAGE-AX induced the apparition of more apoptotic and necrotic areas in tumors than in mice without treatment. These results highlight the properties of GK-1 as an immunostimulant of DCs and suggest as a potential candidate the use of this immunotherapy against cancer disease.

## 1. Introduction

Melanoma is a very aggressive skin cancer due to its high metastatic capacity [1, 2]. Early diagnosis increases the survival rate of 95% at 5 years; however, if the diagnosis is delayed the chance of survival decreases to 5% in a year. Surgery, chemotherapy, radiotherapy, and combinations of these have been used for the treatment of melanoma, with poor results [2, 3]. Thus, in recent years, new possibilities with different immunotherapy approaches have been explored [4], like nonspecific immunomodulation based on the use of various cytokines (IL-2, IL-12, and IFN*γ*), antibodies, and TLR ligands [5–7], in addition to adoptive transfer [8]. Among them, the use of antibodies recognizing CTLA-4 and PD-1 stands out, in an attempt to reverse the inhibition of lymphocyte activation and the adoptive transfer of autologous T cells activated* ex vivo*, although their success has been limited [9–11].

For over a decade, dendritic cells (DCs) have been used in immunotherapy against different varieties of cancer [4, 12, 13]. The most used DCs are the ones differentiated from bone marrow cells or peripheral blood monocytes [14]; particularly the DCs derived from bone marrow cells (BMDCs) may have a greater capacity to activate T lymphocytes [10]. The use of DCs also enables the possibility to load them with tumor peptides and/or with necrotic or apoptotic tumor cells [15, 16]. Although the results are far from satisfactory, the cells loaded with tumor peptides have been proven the most effective in immunotherapy with DCs [17–20]. Also recently, molecules like TNF*α*, IFN*γ*, prostaglandins, alpha galactosylceramides, or TLR ligands have been used. These have increased the activation and antigen-presenting capacity of DCs [21, 22].

Despite the efforts made in the area of immunotherapy, the control of advanced melanoma remains a challenge, so in this work we evaluate the possibility of using immunotherapy through the transfer of DCs pretreated with MAGE-AX. MAGE-AX is a highly immunogenic peptide, composed of eight amino acid residues, common to the MAGE A1-3 and A5 tumor proteins, which are located primarily in tumor cells [23]. Furthermore, in order to increase the efficiency of this treatment, we included a synthetic peptide designated GK-1, which is one of the three components of the S3Pvac vaccine against porcine cysticercosis [24], which has properties that might be useful to increase the antigen-presenting capacity of DCs [24, 25]. Experimental evidence shows that, in addition to inducing a specific immune response, GK-1 possesses an adjuvant capacity that increases the efficiency of influenza vaccine in mice and pigs [25, 26]. On the other hand, it has been shown that coimmunization of GK-1 with the influenza vaccine increases the specific lymphocyte proliferative capacity against influenza induced by an increase in the activity of DCs [25]. Recently, it has been reported that subcutaneous administration of GK-1 in mice with melanoma induces regression of the tumor mass and increases survival. So the aim of this study was to evaluate the capacity of GK-1 as an immunomodulator in DCs loaded with the MAGE-AX peptide for immunotherapy against murine melanoma.

## 2. Materials and Methods

### 2.1. Ethics Statement

The study was approved by the Faculty of Medicine, UNAM ethical review board, and was performed in accordance with the Mexican Official Norm NOM 062-ZOO-1999.

### 2.2. Mice

60 male C57BL/6 (H2K^b^) aged six to eight weeks old mice were included in each experiment. They were kept in controlled light-dark and temperature conditions and fed* ad libitum* in the animal facilities of the Department of Cell and Tissue Biology from the Faculty of Medicine, UNAM.

### 2.3. Reagents

Monoclonal antibodies for staining of cells analyzed by flow cytometry, CD3-biotin, CD8-CyCrome, CD11c-allophycocyanin, CD40-biotin, CD86-biotin, Ia/Ie-phycoerythrin, IL-12-biotin, IFN*γ*-biotin, and IL-10-biotin, were purchased from Pharmingen, BD Bioscience, USA. The B16/F10 murine melanoma cell line with the H-2K^b^ haplotype was acquired from the American Type Culture Collection, USA. The carcinoma cell line of Chinese hamster ovary (CHO) transfected with the granulocyte-macrophage growth factor gene (GM-CSF) was kindly donated by Dr. Edda Sciutto from the Institute for Biomedical Research, UNAM. The RPMI-1640 and F12 (Ham) culture media were purchased from GIBCO, USA.

### 2.4. Peptides

The MAGE-AX (LGITYDGM) protein was synthesized by Research Genetics (Invitrogen, Leiden, Holland), with a 94% purity. The GK-1 peptide (GYYYPSDPNTFYAPPSA), with 96.11% purity, was synthesized by Alpha Diagnostics International, San Antonio, TX, USA. Both peptides were stored at −70°C. Using the Limulus amebocyte lysate (LAL) assay from Thermo Lab, the absence of bacterial endotoxin in both peptides was confirmed.

### 2.5. Differentiation of Dendritic Cells Derived from Bone Marrow (BMDCs)

For the differentiation of BMDCs, the methodology previously described [27, 28] was used with some modifications. The femur and tibia of three C57BL/6 mice were obtained, the muscle was removed, and the bones sanitized by continuous washing with 70% ethanol for 2 min. Thereafter, to obtain the bone marrow, the epiphyses of the bones were removed and the diaphysis perfused with PBS (0.4 g/L potassium phosphate, 0.726 g/L sodium phosphate and 9 g/L sodium chloride, Sigma). The bone marrow cells were inoculated in RPMI-1640 medium supplemented with penicillin (100 U/mL), streptomycin (100 *μ*g/mL, Sigma), L-glutamine (2 mM, Sigma), mercaptoethanol (50 *μ*M), and 10% fetal bovine serum (BioWest, USA), in 75 cm^3^ culture flasks at a concentration of 5 × 10^5^ cells per mL. The complete medium was supplemented at 20% with supernatant of the CHO cell line, and the supernatant contained approximately 200 U/mL of GM-SCF. Every third day the GM-CSF supplemented RPMI medium was replaced. At the tenth day of growth, the cells were treated with 1 *μ*g/mL LPS or 50 ng/mL TNF*α*, 25 mg/mL MAGE-AX, and/or 10 *μ*g/mL GK-1 for 24 hours, so that the following groups were formed: (1) without treatment (WT), (2) LPS, (3) TNF*α*, (4) MAGE-AX, (5) GK-1, (6) TNF*α*/MAGE-AX, (7) TNF*α*/GK-1, and (8) TNF*α*/MAGE-AX/GK-1. The degree of maturation and activation of BMDCs was assessed by monitoring the expression of MHCII, CD86, CD80, and CD40 molecules by flow cytometry.

### 2.6. Phenotype Characterization of BMDCs

The BMDCs phenotype was characterized by flow cytometry. Cell suspensions were stained at 4°C with the following antibodies: CD11c-allophycocyanin, Ia/Ie-isothiocyanate, CD40-biotin, CD80-biotin, CD86-biotin, and IL-12-biotin. The BMDCs treated with LPS, TNF*α*, GK-1, and TNF*α*/GK-1 were incubated with 1 *μ*g/mL Brefeldin A for 4 hours; then the cells were fixed with BD Citofix buffer and permeabilized with BD Perm/Wash buffer from BD Bioscience, USA. Afterwards, the BMDCs were stained with anti-IL-12 and streptavidin conjugated to phycoerythrin. Samples were acquired on a BD Bioscience FACScalibur flow cytometer and analyzed with the Flow Jo software.

### 2.7. Tumor Induction

For melanoma induction 60,000 B16/F10 cells were inoculated subcutaneously in 30 C57BL/6 mice for each of the tests performed. After a week of tumor induction, therapy with BMDCs matured with TNF*α*, loaded with MAGE-AX antigens, and stimulated with GK-1 was carried out.

### 2.8. Immunization Protocol

For each of the different treatments five groups of ten mice each randomly assigned were formed, one received no treatment and four received subcutaneous inoculation of 1 × 10^6^ BMDCs matured with TNF*α*; groups were formed according to presence or absence of GK-1 stimulation and MAGE-AX load, as follows: (1) 300 *μ*L of PBS (WT), (2) BMCDs/TNF*α*, (3) BMDCs/TNF*α*/MAGE-AX, (4) BMDCs/TNF*α*/GK-1 and (5) BMDCs/TNF*α*/MAGE-AX/GK-1. The treatment was administered once a week for three weeks. The experiment was repeated, and data were pooled.

### 2.9. Survival and Tumor Size

From the moment in which the groups of mice received the various treatments, the mice survival was recorded, and every other day the largest diameter of the tumors was measured using a vernier caliper.

### 2.10. Staining of Histological Sections with Hematoxylin and Eosin

The histopathological evaluation of the melanomas was carried out following previously described procedures [29]. The dissected tumors were fixed in Zamboni solution (picric acid saturated solution and 4% buffered formalin) for 24 hours. The fixed tumors were embedded in paraffin afterwards. Subsequently, up to 10 histological sections were performed, of about 5 *μ*m each, which were stained with hematoxylin and eosin for histological examination. The record of the histological study was performed using photomicrographs, showing an area of 1,093,456.9 *μ* ^2^. It is important to mention that photomicrographs were taken in the entire area of each of the 10 histological sections, with a 20x objective on a Nikon Eclipse 80i microscope. In order to obtain the area percentage of cell death in each photomicrograph, the area of cell death (acidophilic regions with abundant cells with pyknotic nuclei) was recorded through the use of the Motic Images Plus 2.0 software.

### 2.11. Cytokine Profile Characterization

In order to identify the type of immune response, 1 × 10^6^ cells/mL of peritumoral lymph nodes, obtained from the groups of mice with different treatments, were stimulated with 25 mg/mL of MAGE-AX for 5 days [30]; then the cultures were treated with 1 *μ*g/mL Brefeldin A (BD Bioscience, USA) for 5 hours. Subsequently, the cells were stained at 4°C with anti-CD3-biotin, anti-CD8-cycrome, and streptavidin-isothiocyanate; afterwards they were fixed with BD Citofix buffer and permeabilized with BD Perm/Wash buffer, all from BD Bioscience, USA. Finally, the cells were stained with anti-IFN*γ* biotin, anti-IL-10 biotin, and phycoerythrin conjugated streptavidin antibodies (BD Bioscience, USA). The samples were acquired on a BD Bioscience FACScalibur flow cytometer and analyzed with the Flow Jo software.

### 2.12. Statistical Analysis

Data are shown as means and SEM. Repeated measures analysis of variance test (ANOVA) and Tukey post hoc test was performed in order to evaluate the significance of the effects of the different treatments. A *P* value < 0.05 was considered statistically significant. All analyzes were performed in the GraphPad Prism 6 software, and all graphs were built with the Sigma Plot 12.3 software.

## 3. Results

### 3.1. GK-1 Induces an Increment in CD86 and IL-12 Expression in BMCDs

**Figure 1:**
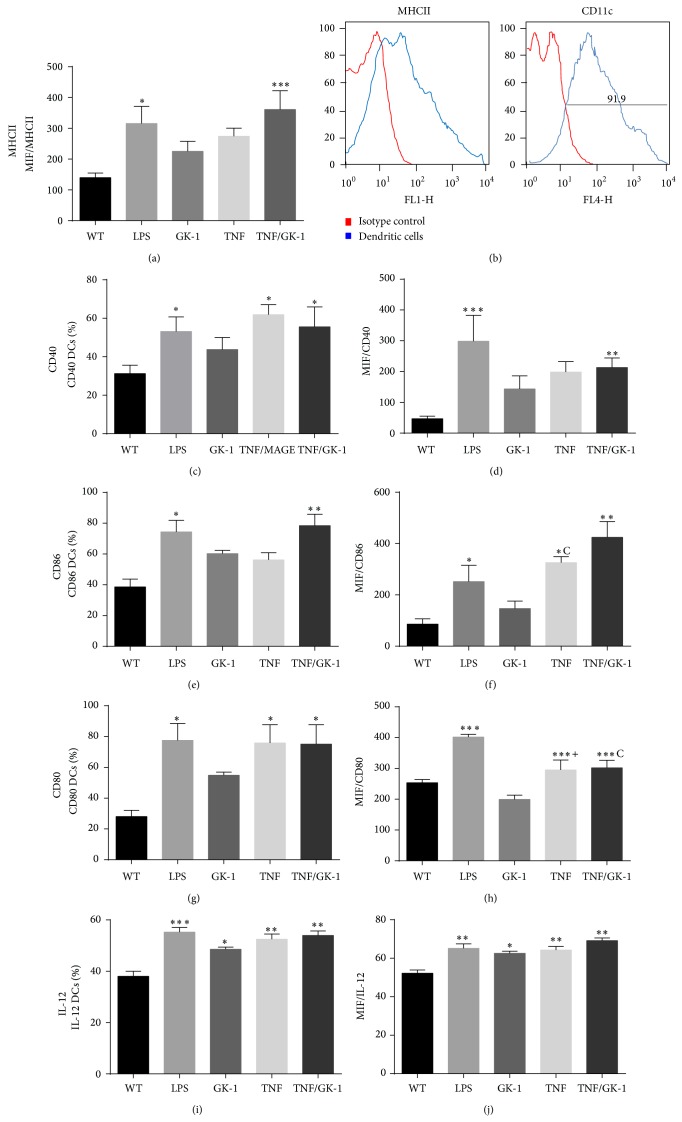
BMDCs phenotype. Levels of molecules of the major histocompatibility complex II (MHCII), CD40, CD80, CD86, and IL-12 in BMDCs were measured after different treatments: control (without treatment: WT), LPS, GK-1, TNF*α*, TNF*α*/GK-1. Treatment with LPS, TNF*α*, and TNF*α*/GK-1 induces increased expression of MHCII, CD40, CD80, and CD86. When BMDCs were treated only with GK-1 an increase in the production of IL-12 was found. (a) Mean fluorescence intensity (MIF) of MHCII. ^*^ *P* < 0.05, ^***^ *P* < 0.001. (b) BMDCs phenotype after 10 days of differentiation. 91.9% differentiation was induced (91.9% of CD11c+ cells). Red: isotype control. Blue: BMDCs. (c) Percentage of CD40+ BMDCs after treatment. ^*^ *P* < 0.05. (d). MFI of CD40 in BMDCs. ^**^ *P* < 0.001, ^***^ *P* < 0.0001. (e) Percentage of CD86+ BMDCs. ^*^ *P* < 0.05, ^**^ *P* < 0.001. (f) MFI of CD86 in BMDCs. ^*^ *P* < 0.05, ^**^ *P* < 0.001, ^c^ *P* < 0.05 TNF*α* versus GK-1. (g) Percentage of CD80+ BMDCs. ^*^ *P* < 0.05. (h) MFI of CD80 in BMDCs. ANOVA, Tukey. ^***^ *P* < 0.0001, ^c^ *P* < 0.0001 TNF/GK-1 versus GK-1, ^+^ *P* < 0.0001 TNF versus GK-1. (i) Percentage of IL-12+ BMDCs. (j) MFI of IL-12 in BMDCs. ^*^ *P* < 0.05, ^**^ *P* < 0.001, ^***^ *P* < 0.0001. Mean ± SEM *n* ≥ 3.

The BMDCs were differentiated from bone marrow cultures of C57BL/6 mice with GM-CSF. 90% of the differentiated cells expressed the CD11c/MHCII+ phenotype (Figure 1(b)).

To corroborate the effect of GK-1 in maturation and activation, the BMDCs were treated with GK-1, TNF*α*, or TNF*α*/GK-1. Treatment with GK-1 induced a slight increase in the expression of molecules of the major histocompatibility complex II (MHCII), CD40, CD80, and CD86; however, only the addition of TNF*α* induced a significant expression (Figure 1). In addition, we analyzed whether GK-1 could induce changes in the percentage of BMDCs positive to MHCII, CD40, CD80, and CD86. The trend percentage of cells positive to MHCII and costimulatory molecules was similar to the trend of the mean fluorescence intensity (MFI). Stimulation with TNF*α* or TNF*α*/GK-1 resulted in a statistically significant increase in the percentage of positive BMDCs to MHCII, CD40, CD80, or CD86 molecules (Figure 1).

**Figure 2:**
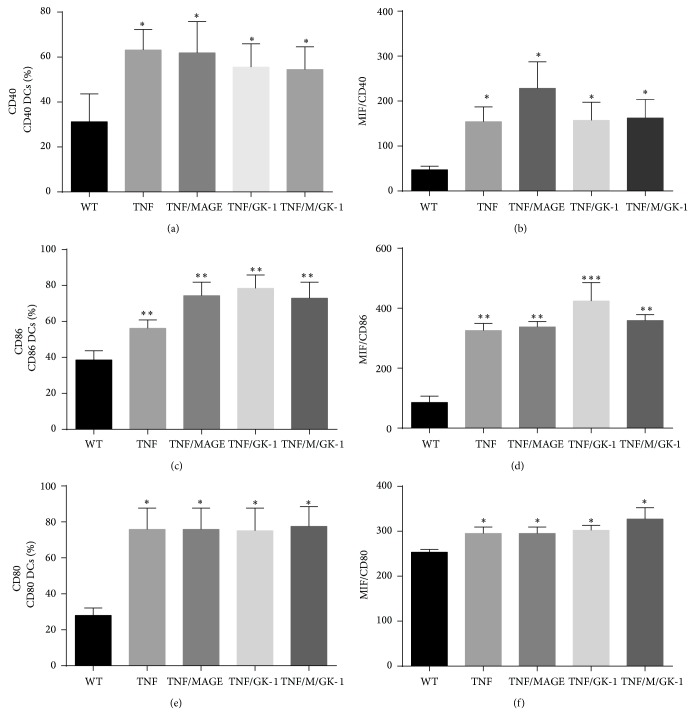
Effect of GK-1 and/or MAGE-AX with TNF*α* in the BMDCs phenotype. Treatment with MAGE-AX did not induce changes in the phenotype of BMDCs. (a) Percentage of CD40+ BMDCs. ^*^ *P* < 0.05. (b) MFI of CD40 in BMDCs. ^*^ *P* < 0.05. (c) Percentage of CD86+ BMDCs. ^**^ *P* < 0.001. (d) MFI of CD86 in BMDCs. ^**^ *P* < 0.05, ^***^ *P* < 0.001. (e) Percentage of CD80+ BMDCs. ^*^ *P* < 0.05. (f) MFI of CD80 in BMDCs. ^*^ *P* < 0.05. Mean ± SEM *n* ≥ 3.

MAGE-AX was used as a tumor antigen to induce a specific response against melanoma, so also it was verified whether exposure to MAGE-AX could induce a change in the expression of MHCII and costimulatory molecules. The BMDCs phenotype matured with TNF*α* with or without GK-1 and MAGE-AX showed no significant changes in the MFI of costimulatory molecules or in the percentage of positive BMDCs to these molecules (Figure 2).

To assess IL-12 production in the BMDCs, the obtained cells were treated with TNF*α*, GK-1, or TNF*α*/GK-1 for 24 hours and then incubated with Brefeldin A for 5 hours and finally IL-12 production was determined. An increase was noted in the expression and number of cells positive to IL-12 in the groups of cells stimulated with TNF*α*, GK-1, and TNF*α*/GK-1 in comparison with the control group (WT). The presence of TNF*α* did not induce a higher production of IL-12 than GK-1 (Figures 1(i) and 1(j)).

### 3.2. Increased Survival and Reduced Tumor Growth Rate in Mice Treated with BMDCs Loaded with MAGE-AX and GK-1 Stimulated

**Figure 3:**
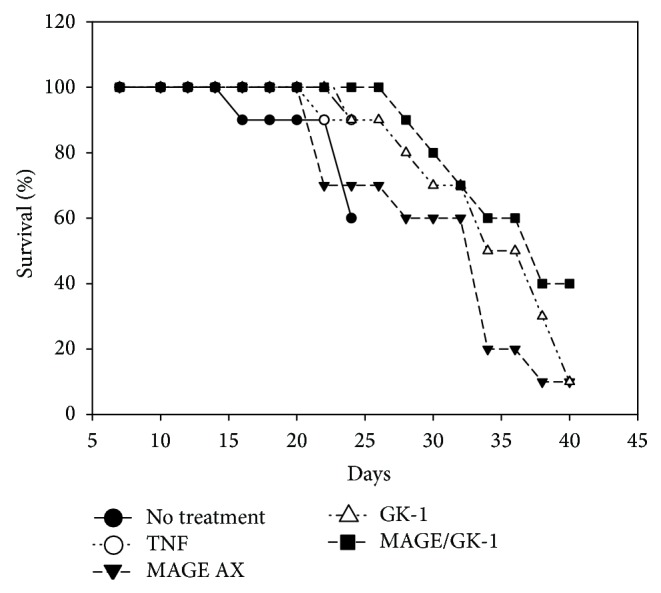
Mice survival. Survival of mice with melanoma inoculated with BMDCs matured with TNF*α* and treated with MAGE-AX, GK-1, or MAGE-AX/GK-1. The MAGE-AX/GK-1 group was the one which had a higher survival rate: 40% up to 1.5 years after being inoculated with the BMCDs. It is followed by the GK-1, MAGE-AX, TNF*α*, and untreated group (WT), respectively.

All BMDCs used in the immunotherapy were matured with TNF*α* and treated with (1) GK-1, (2) MAGE, or (3) MAGE-AX/GK-1. BMDC therapy started one week after inoculation of 6 × 10^5^ B16F10 cells. Mice receiving BMDCs loaded with MAGE-AX and stimulated with GK-1 showed a higher survival rate relative to the control groups. Mice that received no therapy as well as those who received the BMDCs/TNF*α* treatment showed the lowest survival rate (100% death at days 24-25). The BMDCs groups treated with TNF*α*/MAGE-AX or GK-1 had a survival of at least 10% at day 40, while 40% of the mice that received the BMDC treatment with TNF*α*/MAGE-AX/GK-1 achieved a 40% survival rate up to 1.5 years after being inoculated with melanoma (Figure 3).

**Figure 4:**
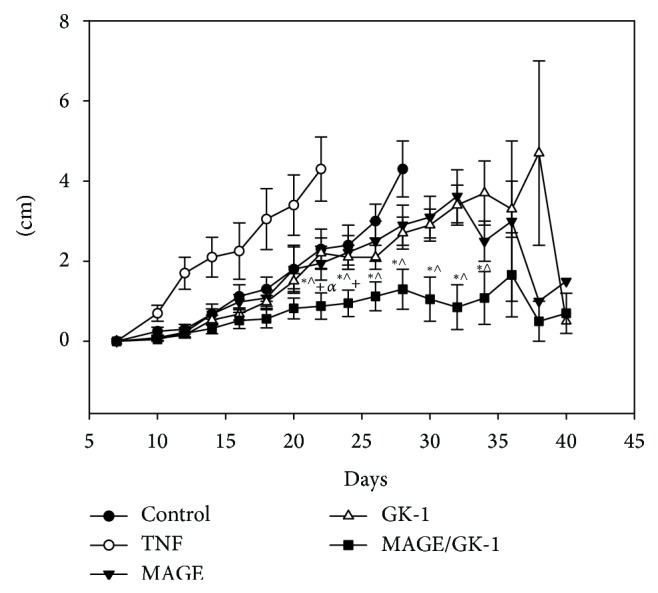
Tumor development. Tumor size in mice inoculated with BMDCs matured with TNF*α* and treated with MAGE-AX, GK-1, or MAGE-AX/GK-1. From day 22 to day 24 the group treated with MAGE/GK-1 BMDCs had less tumor growth in comparison with all groups. From day 26 no tumor growth was shown in the TNF*α* and untreated groups, because the survival rate was 0%. Tumor growth of the MAGE-AX and GK-1 groups was similar. Without treatment (WT). Mean ± SEM *n* ≥ 3. ^*^ *P* < 0.05 MAGE/GK-1 versus MAGE, ^∧^ *P* < 0.05 MAGE/GK-1 versus GK-1, ^+^ *P* < 0.001 MAGE/GK-1 versus TNF, ^*α*^ *P* < 0.001 MAGE/GK-1 versus WT.

On the other hand, the largest diameter of the tumor was measured every other day. The groups treated with TNF*α*/MAGE-AX or GK-1 BMDCs remained similar in size throughout the treatment, whereas untreated mice and those inoculated with TNF*α* BMDCs showed an increased tumor growth rate compared to the other groups. It is important to note that the group of mice that received TNF*α*/MAGE-AX/GK-1 BMCDs retained the lowest rate of tumor growth since the beginning of the treatment (Figure 4).

### 3.3. GK-1 Stimulated BMDCs Induced an Increase in the IFN*γ* and IL-10 Production for CD8 Lymphocytes from Lymph Nodes

**Figure 5:**
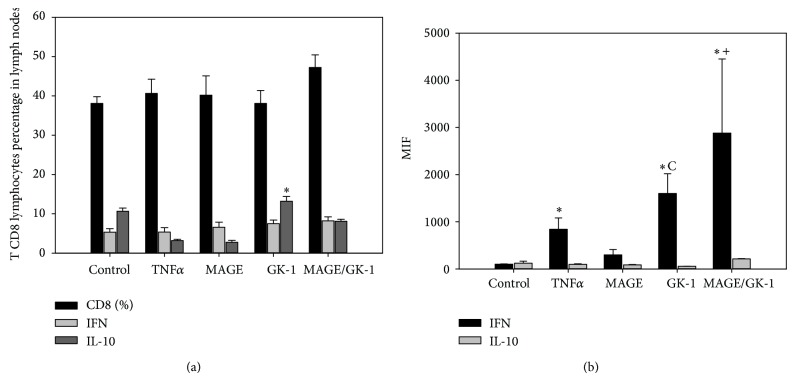
Cytokine profile. Percentage of total CD8 T cells and IFN*γ* or IL-10 producing CD8 cells, in peritumoral lymph nodes of mice with melanoma inoculated with BMDCs matured with TNF*α* and treated with MAGE-AX, GK-1, or MAGE-AX/GK-1. Mice treated with BMDCs MAGE/GK-1 showed increased levels of IFN*γ* in CD8+ lymphocytes. (a) Percentage of total CD8 T cells and IFN*γ* and IL-10 producers. ^*^ *P* < 0.05 GK-1 versus all groups. (b) MFI of IFN*γ* and IL-10 in CD8 T lymphocytes. ^*^ *P* < 0.05, ^c^ *P* < 0.05 GK-1 versus MAGE, ^+^ *P* < 0.05 MAGE/GK-1 versus MAGE. Without treatment (WT). Mean ± SEM *n* ≥ 3.

No significant differences were found in the percentage of CD8 T lymphocytes in lymph nodes peripheral to the tumor (Figure 5(a)). In terms of cytokine production, in CD8 T lymphocytes, the TNF*α*, TNF*α*/GK-1, and TNF*α*/MAGE-AX/GK-1 groups showed increased production of IFN*γ* compared to the WT group and TNF*α*/MAGE-AX BMDCs; however, groups treated with TNF*α*/MAGE-AX/GK-1 and TNF*α*/GK-1 BMDCs showed a higher IFN*γ* production than the TNF*α* group (Figure 5(b)). Finally, in the case of IL-10, the GK-1 group showed a significant increase in the percentage of CD8 IL-10+ T cells in comparison to the TNF*α*/MAGE-AX, TNF*α*/GK-1, and TNF*α*/MAGE-AX/GK-1 groups (Figure 5(a)). No changes were observed in the IL-10 MFI (Figure 5(b)). It is important to note that the higher levels of fluorescence were showed by IFN*γ* in CD8+ T lymphocytes (Figure 5(b)), in comparison with IL-10, although the group treated with TNF*α*/GK-1 BMDCs showed a significant increase in the percentage of CD8 IL-10+ T lymphocytes in comparison to the other groups (Figure 5(a)).

### 3.4. Histopathological Examination Showed That the Administration of GK-1 Stimulated BMDCs Induced an Increase in the Areas of Tumor Cell Death

**Figure 6:**
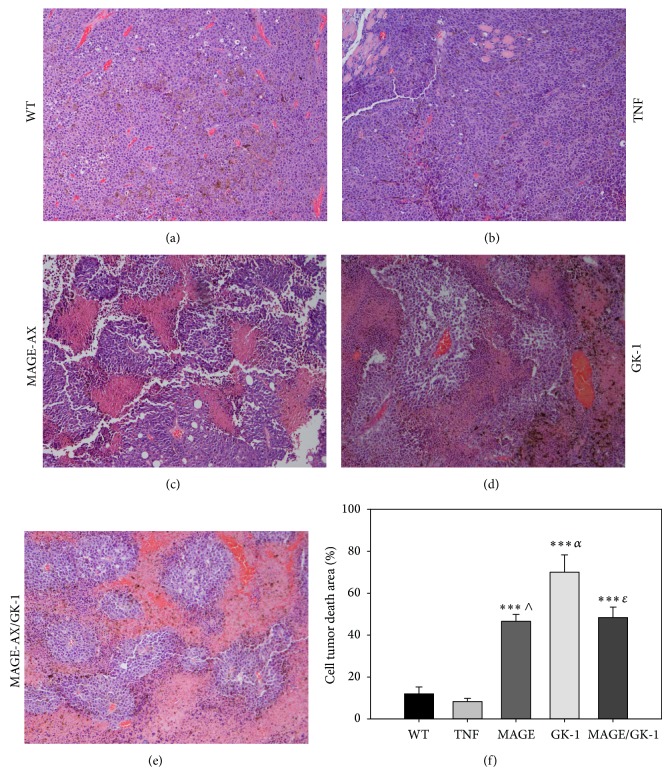
Histopathological analysis. Photomicrographs of histological sections of tumors from mice vaccinated with BMDCs matured with TNF*α* and treated with MAGE-AX, GK-1, or MAGE-AX/GK-1. Groups MAGE-AX, GK-1, and MAGE-AX/GK-1 showed plentiful tumor cell death areas. (a) No treatment (WT). Abundant tumor cells and blood vessels were observed. (b) TNF*α*. The same characteristics as those observed in untreated mice were observed: abundant tumor cells and blood vessels. (c) MAGE-AX. (d) GK-1. (e) MAGE-AX/GK-1. In (c), (d), and (e), pink areas (eosinophilic), composed of dead cells, were observed in addition to purple areas (basophilic), composed of very active tumor cells. (f) A graph showing the change in the areas of cell death in tumors from mice that were treated with BMDCs and which were stimulated with TNF*α*, MAGE-AX, GK-1, or MAGE-AX/GK-1. ^***^ *P* < 0.001, ^∧^ *P* < 0.001 MAGE versus TNF, ^*α*^ *P* < 0.0001 GK-1 versus TNF*α*, ^*ɛ*^ *P* < 0.001 MAGE/GK-1 versus TNF. Mean ± SEM *n* ≥ 3.

The histopathological examination of the tumors of the WT mice and from those who received BMDCs treated with TNF*α* showed abundant epithelioid cell nests, characterized by the presence of many nuclei with abundant euchromatin, which is indicative of a great cellular activity. Also numerous blood vessels between the nests of epithelioid cells were observed (Figures 6(a)-6(b)). Both parameters are related to tumors with a high growth rate [29]. While sections of the tumors from mice that received treatment with MAGE-AX, GK-1, or MAGE-AX/GK-1 BMDCs showed numerous areas of cell death and fewer areas with epithelioid cells than control group, the areas of cell death were characterized by showing eosinophilic regions formed by cellular debris and cells with pyknotic nuclei (Figures 6(c), 6(d), and 6(e)). Afterwards, photographs were taken of the total tumor area, in order to evaluate the percentage of the area of cell death in the tumors (Figure 6(f)). It was observed that the tumors from WT mice and from those who received treatment with TNF*α* BMDCs were solid tumor masses with few areas of cell death, whereas the tumors from the mice that received therapy with the TNF*α*/MAGE-AX, TNF*α*/GK-1, or TNF*α*/MAGE-AX/GK-1 BMDCs showed larger cell death areas than those from the mice in the control groups and those that received TNF*α* BMDCs. No significant differences between the TNF*α*/MAGE, TNF*α*/GK-1, and TNF*α*/MAGE/GK-1 groups were found (Figure 6(f)).

## 4. Discussion

In the present study we show that BMDCs loaded with MAGE-AX significantly decrease the rate of tumor growth through an increase in the specific antitumor immune response against induced melanoma (Figures 4 and 5), which results in an increase of the area of cell death in tumors (Figure 6) and in the survival time of mice with melanoma (Figure 3). Furthermore, the additional treatment with GK-1 of BMDCs loaded with MAGE-AX significantly increased the efficiency of the immunotherapy.

The effect of GK-1 on the expression of activation markers and BMDCs maturation deserves special comment. GK-1 significantly increased CD86 expression and IL-12 production in BMDCs (Figure 1). CD86 is a molecule involved in the immunological synapse; therefore, the increase can be seen as an increase in antigen-presenting capacity of BMDCs that results in the activation of specific T cells against tumors [22, 28, 31, 32]. On the other hand, IL-12 can induce the development of a Th1 response as well as an increase in IFN*γ* production. Both IL-12 and IFN*γ* are involved in immunosurveillance against tumor cells [33–36]; likewise, it has been shown that exogenous administration of IL-12 in mice with induced tumors may promote the reduction in tumor size by increasing the activation of CD8+ T lymphocytes, which are responsible for inducing apoptosis in tumor cells [37, 38]. The use of BMDCs with a mature phenotype and the ability to produce IL-12 is vital for effective results in antitumor immunotherapy, since it has been demonstrated that immature DCs, characterized by low expression of costimulatory molecules, can induce a tumor immune nonresponse, tolerance, or even absence of immunological memory in patients with melanoma [39]. Furthermore, the use of mature and active DCs can induce immunological memory as well as an effective immune response characterized by a CD8 T lymphocytes-dependent response [40, 41].

In this work, it was observed that GK-1 positively modulated BMDCs activation by inducing an increased expression of CD86 and IL-12. This may explain the decrease in the rate of tumor growth, the development of a Th1 response, and the cell death observed in the histopathology of this study. GK-1 is a molecule whose physicochemical properties can improve its recognition by scavenger receptors on the membrane of BMDCs, promoting tumor antigen presentation and releasing proinflammatory cytokines, situation already observed in DCs treated with GK-1 [42]. Furthermore, the obtained results increase the likelihood of the use of immunotherapy mediated by DCs transfer and the use of immunomodulators, such as GK-1, in both curative and preventive controls of melanoma in those individuals who have had their tumor resected.

Even though GK-1 stimulated BMDCs induced an increase in the antitumor immune response, the use of MAGE-AX BMDCs produced a specific response against the tumor of almost the same intensity as that induced by GK-1. Furthermore, it is noteworthy that the addition of MAGE-AX to BMDCs cultures caused no adverse changes in cell activation (Figure 2); therefore, BMDCs could be used in immunotherapy. MAGE antigens have been used already in immunotherapy; the results have been mixed, since the developed immune response has not been effective and tumor regression has been sporadic. However, when DCs loaded with MAGE-AX were used, there has been increase in survival, a reduction of tumor size in mice with melanoma, and development of a specific immune response dependent on CD8 T cells and IFN*γ* production [23, 43]. Furthermore, MAGE antigens can be found in other types of cancer, like bladder cancer, leukemia, and glioblastoma. However, MAGE-AX is a molecule that can be located in mice, which may be the reason why it would be important to develop a molecule with a consensus sequence in relation to MAGE human molecules, in order to be used in cancer immunotherapy in patients with melanoma or other types of cancer. Therefore, MAGE is a good choice for use in immunotherapy, since it is able to induce a strong specific immune response and can be used in the treatment against various types of cancer [43].

To evaluate the effect of BMDCs transfer in specific antitumor immunity, the levels of CD8+ T lymphocytes in lymph nodes of treated and untreated mice with melanoma were measured (Figure 5). The route of BMDCs inoculation was subcutaneous, so that the response was studied in peritumoral lymph nodes, as it has been studied that the subcutaneous administration of DCs induced a Th-1 response by lymphocytes from peritumoral lymph nodes [44], while the intravenous administration of DCs induces a response dependent on spleen lymphocytes [45, 46]. In our model, a response was observed in the peritumoral lymph nodes as discussed below.

The lymph nodes of the mice that received treatment with BMDCs were analyzed to study the response developed by the treatment. The TNF*α*/MAGE-AX/GK-1 group showed the highest levels of CD8+ T lymphocytes as compared to the other groups (Figure 5(a)). This is important, since a successful antitumor immune response is mediated by T lymphocytes [43, 47]. CD8+ T lymphocytes are responsible for inducing tumor cell death and the secretion of cytokines, which can induce the activation of both T lymphocytes and other immune cells [13, 46]. Therefore, the use of GK-1 contributed to the increase in the antitumor response mediated by T cells located in lymph nodes, whereas MAGE-AX promoted that such response was specific against melanoma.

Our data demonstrates there was increased amount of T CD8 IL-10+ lymphocytes and IFN*γ* MIF when mice with melanoma were treated with BMDCs stimulated with GK-1 and loaded with MAGE-AX. It is highly remarkable to mention that although the percentage of CD8 IL-10+ was increased, the MIF of IFN*γ* was higher than IL-10 MIF in T CD8 lymphocytes. IFN*γ* is a cytokine associated with a Th1 antitumor effective immune response, characterized by proliferation of cytotoxic CD8+ T cells and macrophage activation [37, 48, 49]. It is possible that the increase of IFN*γ* has been induced by IL-12 secretion by BMDCs [38], since it was observed that GK-1 stimulated the increase in the production of this cytokine (Figures 1(i) and 1(j)). It is known that IL-12 can stimulate T cells to produce IFN*γ* [33, 38, 50], so these events also could have happened in our model.

In the case of IL-10, several studies have reported that patients with cancer present high IL-10 concentrations, which may be the reason why its increment has been correlated with a poor prognosis [51]. It has also been described that this cytokine induces an immunosuppressive tumor environment, resulting in the inhibition of cells such as macrophages, DCs, and lymphocytes [52]. It is therefore noteworthy that BMDCs loaded with MAGE-AX and stimulated with GK-1 did not induce significant changes in IL-10, but they promoted high IFN*γ* levels in CD8+ T lymphocytes in peritumoral lymph nodes (Figure 5), vital signs for an effective Th1 antitumor response to be carried out, which is hoped for in cancer patients.

The aforementioned data were correlated with the histopathological findings, the survival and the tumor growth rate. Treatment with the MAGE-AX/GK-1 BMDCs showed tumors with abundant areas of cell death (Figure 6), low tumor growth (Figure 4), and a survival rate of up to 1.5 years after the melanoma being induced (Figure 3). It is important to remember that in the lymph node of mice receiving TNF*α*/MAGE-AX/GK-1 BMDCs, increased levels of CD8 T lymphocytes were found, which are involved in the induction of tumor cell death [9, 30], so it is very likely that the increase in the area of cell death as well as the decrease in tumor growth and survival gain were caused by the activation of CD8 T cells. On the other hand, it has been studied that tumor cell death also increases the immune response against the tumor [5]; therefore it is possible that cell death induced by the vaccination with BMDCs loaded with MAGE-AX and stimulated with GK-1 helps to increase the antitumor immune response in this model.

## 5. Conclusions

Our findings demonstrate that the addition of GK-1 to BMDCs loaded with MAGE-AX decreases the rate of tumor growth and increases the survival of mice and the expression of CD86 and IL-12 in the BMDCs, as well as the levels of CD8 IFN*γ* producing T cells. The presence of tumor cell death is also evident, indicating that GK-1 may positively immunomodulate a Th1 response that favors tumor growth control. The evidence reported in this study indicates the usefulness of GK-1 not only for the treatment of melanoma, but also preventively, as in the case of people who have undergone cancer surgery and want to prevent the reappearance of a tumor. It is also appropriate to investigate whether GK-1 may be involved in the activation of other cell types associated with immunosurveillance, so that it can be used in the activation of other cell lines that could be used in immunotherapy in the future.

## Abbreviations

IL: Interleukin
IFN*γ*: Interferon gamma
TNF*α*: Tumor necrosis factor alpha
TLR: Toll like receptor
CTLA-4: Cytotoxic T-lymphocyte antigen 4
DCs: Dendritic cells
BMDCs: Bone marrow dendritic cells
MAGE-AX: Melanoma-associated antigen AX
GM-CSF: Granulocyte macrophage colony-stimulating factor
MIF: Mean fluorescence intensity.
