# Corrigendum to “GK-1 Improves the Immune Response Induced by Bone Marrow Dendritic Cells Loaded with MAGE-AX in Mice with Melanoma”

**DOI:** 10.1155/2015/176840

**Published:** 2015-12-28

**Authors:** Gabriela Piñón-Zárate, Miguel Ángel Herrera-Enríquez, Beatriz Hernández-Téllez, Katia Jarquín-Yáñez, Andrés Eliú Castell-Rodríguez

**Affiliations:** Laboratorio de Inmunoterapia e Ingeniería de Tejidos, Departamento de Biología Celular y Tisular, Facultad de Medicina, Universidad Nacional Autónoma de México Edificio A, Sexto Piso, Ciudad Universitaria, Avenida Universidad No. 3000, Ciudad de México 04510DF, Mexico

In the paper titled “GK-1 Improves the Immune Response Induced by Bone Marrow Dendritic Cells Loaded with MAGE-AX in Mice with Melanoma” [1] the phrase “Recently, it has been reported that subcutaneous administration of GK-1 in mice with melanoma induces regression of the tumor mass and increases survival” should be as follows: “Recently, our group has shown that subcutaneous administration of GK-1 and DCs in mice with melanoma induces regression of the tumor mass and increases survival (unpublished data).”
